# Diagnostic and Epitope Mapping Potential of Single-Chain Antibody Fragments Against Foot-and-Mouth Disease Virus Serotypes A, SAT1, and SAT3

**DOI:** 10.3389/fvets.2020.00475

**Published:** 2020-08-11

**Authors:** Melanie Chitray, Pamela Anne Opperman, Lia Rotherham, Jeanni Fehrsen, Wouter van Wyngaardt, Janine Frischmuth, Elizabeth Rieder, Francois Frederick Maree

**Affiliations:** ^1^Agricultural Research Council, Onderstepoort Veterinary Research, Vaccines and Diagnostic Development, Onderstepoort, Pretoria, South Africa; ^2^Department of Veterinary Tropical Diseases, Faculty of Veterinary Science, University of Pretoria, Pretoria, South Africa; ^3^Department of Production Animal Studies, Faculty of Veterinary Science, University of Pretoria, Pretoria, South Africa; ^4^Biotechnology Division, National Bioproducts Institute, Pinetown, South Africa; ^5^Plum Island Animal Disease Centre, U.S. Department of Agriculture, Agricultural Research Service, Greenport, NY, United States; ^6^Department of Biochemistry, Genetics and Microbiology, Faculty of Natural and Agricultural Sciences, University of Pretoria, Pretoria, South Africa

**Keywords:** foot-and-mouth disease, SAT1, SAT3, serotype A, phage display, single-chain variable fragment, epitope, ELISA

## Abstract

Foot-and-mouth disease (FMD) affects cloven-hoofed domestic and wildlife animals and an outbreak can cause severe losses in milk production, reduction in meat production and death amongst young animals. Several parts of Asia, most of Africa, and the Middle East remain endemic, thus emphasis on improved FMD vaccines, diagnostic assays, and control measures are key research areas. FMD virus (FMDV) populations are quasispecies, which pose serious implications in vaccine design and efficacy where an effective vaccine should include multiple independent neutralizing epitopes to elicit an adequate immune response. Further investigation of the residues that comprise the antigenic determinants of the virus will allow the identification of mutations in outbreak strains that potentially lessen the efficacy of a vaccine. Additionally, of utmost importance in endemic regions, is the accurate diagnosis of FMDV infection for the control and eradication of the disease. To this end, a phage display library was explored to identify FMDV epitopes for recombinant vaccines and for the generation of reagents for improved diagnostic FMD enzyme-linked immunosorbent assays (ELISAs). A naïve semi-synthetic chicken single chain variable fragment (scFv) phage display library i.e., the *Nkuku*^®^ library was used for bio-panning against FMD Southern-African Territories (SAT) 1, SAT3, and serotype A viruses. Biopanning yielded one unique scFv against SAT1, two for SAT3, and nine for A22. SAT1 and SAT3 specific scFvs were exploited as capturing and detecting reagents to develop an improved diagnostic ELISA for FMDV. The SAT1 soluble scFv showed potential as a detecting reagent in the liquid phase blocking ELISA (LPBE) as it reacted specifically with a panel of SAT1 viruses, albeit with different ELISA absorbance signals. The SAT1svFv1 had little or no change on its paratope when coated on polystyrene plates whilst the SAT3scFv's paratope may have changed. SAT1 and SAT3 soluble scFvs did not neutralize the SAT1 and SAT3 viruses; however, three of the nine A22 binders i.e., A22scFv1, A22scFv2, and A22scFv8 were able to neutralize A22 virus. Following the generation of virus escape mutants through successive virus passage under scFv pressure, FMDV epitopes were postulated i.e., RGD+3 and +4 positions respectively, proving the epitope mapping potential of scFvs.

## Introduction

Diseases caused by RNA viruses are often difficult to control because of the high mutation rate and the continual emergence of novel genetic and antigenic variants that allow escape from immunity (1). Antigenic differences between viruses play a role in whether the immunity induced by one virus is effective against another. Foot-and-mouth disease (FMD) virus (FMDV), a single-stranded, positive-sense RNA virus, and the prototype member of the *Aphthovirus* genus in the family *Picornaviridae* (2), is an example of an antigenically variable pathogen with the ability to evade the immune system (3–5). Of the seven clinically indistinguishable FMDV serotypes, viruses belonging to the three Southern African Territories (SAT) serotypes display appreciably greater genomic and antigenic variation (6).

Two key research focus areas for enhanced FMD control are improved vaccines that offer a broad immunogenic response and improved specific diagnostic assays (7). However, the high antigenic diversity that exists within the FMDV serotypes hinders FMD control by vaccination, as vaccination against one serotype does not confer protection against another and may only be partially effective against some subtypes within the same serotype (8). This poses serious implications in vaccine design and efficacy where an effective vaccine should include multiple independent epitopes to elicit an immune response (9). The humoral immune response has generally been accepted as the most important factor in conferring vaccine-induced protection against FMD, as a strong correlation has been reported between the levels of virus-neutralizing antibody produced after vaccination and subsequent protection of cattle, one of the main target species for vaccination (10–13). To develop more effective vaccines or peptide vaccines, numerous FMDV studies have been undertaken to identify these neutralizing antigenic sites in more detail (14). Neutralizing antigenic sites have been identified for serotype A (15–17), O (18–21), C (22), Asia-1 (23), and SAT2 (19, 24, 25). However, information regarding the antigenic determinants of SAT serotypes, which are confined geographically to Africa, is scarce (26). Mapped SAT2 epitopes include: (i) βG–βH loop of VP1; (ii) residue 210 in the C-terminus of VP1; (iii) VP1 84–86, 109–111, VP2 71, 72, 133, 134; and (iv) VP1 159, VP2 71–72, 133–134, 148–150 (19, 24, 25, 27, 28). Four independent antigenic determinants were identified for SAT1 viruses i.e., (i) two occurring in the βG–βH loop of VP1; (ii) two simultaneous residues one in VP3 (position 135 or 71 or 76) and one in VP1 (position 179 or 181); (iii) a conformation dependant site within VP1 position 181 and VP2 72; and (iv) VP1 position 111 (24). To date, no neutralizing sites have been determined for viruses of the SAT3 serotype. It has been shown that the majority of FMDV-neutralizing antibodies are directed against conformational epitopes located on the β-barrel connecting loops, especially the highly mobile βG–βH loop in VP1 (15, 18, 26, 29, 30). Therefore, knowledge of the amino acid residues that comprise the antigenic determinants of FMDV, and those that function as protective epitopes in particular, will greatly improve our understanding of virus neutralization *in vivo* (12, 26, 31).

Diagnostic assays hampered by the lack of specificity caused by polyclonal capture and detection antibodies highlighted the need for more specific tests. Monoclonal antibodies are highly specific reagents and are being used for a variety of research and diagnostic purposes within the FMD field and their pivotal role in all aspects of FMD research is now clear. However, traditional monoclonal antibodies, produced using hybridoma technology, and used in diagnostics have several limitations such as its high cost, time-consuming production, and the expertise required (32–34).

The development of large combinatorial antibody libraries based on antibody genes expressed and displayed on phages have revolutionized the selection and isolation of unique antibodies to an antigen and aided in the development of recombinant reagents for ELISA (35). A key advantage of phage display of antibody fragments is that the generation of specific single-chain variable fragment (scFv) or antigen binding fragment (Fab) to a particular antigen can be completed within a few weeks compared to hybridomas taking months. Antibody libraries can be either immune (from immunized donors) or naïve (from non-immunized donors). An immune library will have an antibody range that is highly enriched for antibodies generated in response to a particular immunogen, whereas, the naïve library can advantageously be used for an unlimited array of immunogens (36).

Phage display libraries have been used with success to map epitopes for FMDV for serotype O (37, 38) and the SAT2 serotype (25). The *Nkuku*^®^ phage-display library, which is a large semi-synthetic library of recombinant filamentous bacteriophages displaying scFv's derived from combinatorial pairings of chicken variable heavy and light chains, was used for this study (36). This naïve library has been utilized to generate a variety of antibodies against antigens such as the bluetongue virus, African horse sickness virus, echovirus 1, coxsackievirus B3, FMDV of the SAT2 serotype as well as a mycobacterial 16 kDa antigen (25, 36, 39–42). These studies prove that this library is sufficiently diverse for the recognition of a variety of different haptens, proteins, bacteria, and viruses.

In this study, phage display technology was used to obtain specific scFvs from panning with FMDV serotype A, SAT1, and SAT3 viruses. This is a novel study as recombinant monoclonal antibodies (scFvs) have not been isolated for FMDV serotype A, SAT1, and SAT3. The scFvs resulting from the biopanning were investigated in virus neutralization assays in the pursuit of epitope identification and for their prospective use as FMDV diagnostic reagents in an ELISA.

## Materials and Methods

### Cell Cultures, Virus Propagation, and Purification

Baby hamster kidney (BHK) strain 21 clone 13 cells (ATCC CCL-10), used for virus propagation and SAT1 and SAT3 serotype neutralization assays, were maintained in Glasgow minimum essential medium (GMEM, Sigma-Aldrich), supplemented with 10% (v/v) fetal bovine serum (FBS, Hyclone), 1 × antibiotic-antimycotic solution (Invitrogen), 1 mM L-glutamine (Invitrogen), and 10% (v/v) tryptose phosphate broth (TPB, Sigma-Aldrich. Instituto Biologico Renal Suino-2 (IBRS-2) cells used for A22 virus propagation and virus neutralization tests, were maintained in Roswell Park Memorial Institute (RPMI) medium (Sigma-Aldrich) supplemented with 10% (v/v) fetal bovine serum (FBS, Hyclone) and 1 × antibiotic-antimycotic solution (Invitrogen). The Mycl-9E10 hybridoma (ECACC 85102202) was cultured in protein-free hybridoma medium (Invitrogen).

The SAT1/KNP/196/91 and SAT3/KNP/10/90 FMDVs used for the biopanning, originated from buffalo in the Kruger National Park (KNP) in South Africa, isolated during 1991 and 1990, respectively (Table 1). Also used for biopanning was the A22 virus (Table 1), which was obtained from the Pirbright Institute, UK. The SAT1 and SAT3 viruses were propagated on BHK-21 cells whilst A22 was propagated on IB-RS-2 cells prior to sucrose density gradient (SDG) purification of 146S particles. Virus particles were concentrated with 8% (w/v) polyethylene glycol (PEG)-8000 (Sigma-Aldrich) and purified on 10–50% (w/v) sucrose density gradients, prepared in TNE buffer (50 mM Tris pH 7.4, 150 mM NaCl, 10 mM EDTA), as described by Knipe et al. (43). Peak fractions corresponding to 146S virion particles (extinction coefficient E259 nm [1%] = 78.8) were pooled and the amount of antigen (μg) was calculated as described previously (44). In a similar way, viruses utilized for the ELISA assays (Table 1) were PEG concentrated, where virus particles were concentrated with 8% (w/v) PEG-8000 (Sigma-Aldrich) and the resulting precipitated pellet was re-suspended in TNE buffer.

**Table 1:** Detailed list of FMDV SAT1, SAT2, SAT3, and A viruses used in this study.

**FMDV Serotype**	**Virus strain**	Passage history^*^	**Genbank accession number**
SAT1	KNP/196/91	PK1RS5	DQ009716
	KNP/3/03	PK1RS1	KJ999914
	SAR/33/00	PK1RS2	KJ999908
	BOT/1/06	PK1RS1	KJ999919
	SAR/9/03	PK1RS1	KJ999911
	ZIM/14/98	BTY2RS2	KJ999925
	SAR/2/10	PK1RS2	KJ999913
	ZAM/2/93	PK1RS3	DQ009719
	KNP/10/03	PK1RS2	KJ999916
	SAR/9/81	B1BHK4B1RS2	DQ009715
	NAM/272/98	PK2RS1	KJ999921
SAT3	KNP/10/90	PK2RS2	KF647849
	KNP/14/96	PK1RS1	MK415741
	SAR/1/06	BHK5 BTY1	MK415736
	KNP/8/02	PK2	MK415739
	BOT/6/98	BTY1RS2	MK415742
	KNP/2/03	PK1RS1	MK415738
	KNP/1/03	PK1RS1	MK415737
	SAR/14/01	PK1RS2	MK415740
	ZAM/5/93	PK1RS4BHK6	MK415744
	ZIM/5/91	BTY1RS4	MK415745
	KNP/6/08	PK1RS1	MK415735
	ZIM/11/94	BTY2RS5	MK415743
SAT2	ZIM/7/83	B1BHK5B2RS2	DQ009726
A	A22/IRAQ	B2/TBTY2BHK2RS2	AY593764
	A24/CRUZEIRO	B6BHK2RS3BHK3	AJ251476

* *PK, pig kidney cells; RS, Instituto Biologico Renal Suino-2 (IB-RS-2) cells; BTY, bovine thyroid cells; B, Bovine; BHK, baby hamster kidney cells*.

### Selection of scFvs Against SAT1/KNP/196/91, SAT3/KNP/10/90, and A22

Selection of virus-specific scFvs from the *Nkuku*^®^ phage display library was performed as described by van Wyngaardt et al. (36) and Opperman et al. (25). Briefly, 2-ml immunotubes (Nunc^®^ Maxisorp), after being coated overnight with purified virus (30 μg/ml) were blocked with 1 × PBS containing 2% (w/v) milk powder (Elite) and incubated with the *Nkuku*^®^ library phage particles (10^12^-10^13^ transducing particles). Exponentially growing *Escherichia coli* TG1 cells (Stratagene, USA) were infected with eluted phage-displayed scFvs that had bound to the specific viruses before plating on TYE plates (15 g/l agar, 8 g/l NaCl, 10 g/l tryptone, 5 g/l yeast extract) supplemented with 2% (w/v) glucose and 100 μg/ml ampicillin. Subsequent to overnight incubation, the bacteria were collected and the phagemids rescued by the addition of M13KO7 helper phage. Infected bacterial cells were incubated overnight in 2 × TY medium (16 g/l tryptone, 10 g/l yeast extract, 5 g NaCl/l) containing 100 μg/ml ampicillin and 25 μg/ml kanamycin. Phages displaying scFvs were precipitated from the cell-free culture supernatant with one-fifth of the original culture volume of 20% (w/v) PEG-8000 in NaCl and were then suspended in 1 × PBS for use in the next selection round. A total of three such selection rounds were performed. The input and output phages from each selection round was titered to monitor enrichment. The outputs of each consecutive selection round was tested in a polyclonal ELISA. Single clones from the third selection round was tested as soluble scFvs for specific binding to SAT1/KNP/196/91, SAT3/KNP/10/90, or A22.

### Polyclonal Phage ELISA

van Wyngaardt et al. (36) and Opperman et al. (25) described the polyclonal phage ELISA. In short, SDG purified virus (30 μg/ml) of either SAT1/KNP/196/91, SAT3/KNP/10/90, or A22 was used to coat 96-well Maxisorp immunoplates (Nunc^®^) overnight at 4°C. To confirm the specificity of the phage-displayed scFvs to the respective viruses, 1 × PBS containing 2% milk powder (Elite) was used as a blocking reagent and negative control. Bound PEG-precipitated phage-displayed scFvs, produced at each selection round, were detected with the MAb B62-FE2 (100 ng/ml, Progen Biotechnik) and horseradish peroxidase-conjugated polyclonal rabbit anti-mouse IgG (PO260, Dako). After a final wash step, substrate/chromogen solution consisting of 4 mM 3,3′,5,5′-Tetramethylbenzidine (Sigma-Aldrich) in substrate buffer (0.1 M citric acid monohydrate, 0.1 M tri-potassium citrate, pH 4.5) and 0.015% H_2_O_2_ was added for the colormetric reaction. Following 10 min incubation at room temperature, the color reaction was stopped with 1 M H_2_SO_4_ and the absorbance values were recorded at an absorbance of 450 nm (A_450nm_).

### Monoclonal Phage ELISA

Following the third round of panning, monoclonal phage antibodies were screened by randomly selecting individual clones from the titration plates and inoculating it into a 96-well tissue-culture plate (Nunc^®^) containing 2 x TY medium supplemented with 100 μg/ml ampicillin and 2% (w/v) glucose. The bacteria were grown overnight with shaking at 30°C. Using a 96-well inoculation device (Sigma-Aldrich: Cat. No. R-2508), bacterial cells were transferred from the overnight plate to a second plate containing 150 μl of fresh medium per well-followed by incubation for 2.5 h at 37°C with shaking. Subsequently, 50 μl of medium that contained 2 × 10^9^ of the M13K07 helper phage was added to each well and the plate incubated for 30 min at 37°C without shaking. Thereafter, plates were centrifuged at 600 × g for 10 min, the supernatant fractions were removed and replaced with 150 μl of 2 × TY medium containing 100 μg/ml ampicillin and 25 μg/ml kanamycin prior to incubation overnight at 30°C with shaking. Following centrifugation at 600 × g for 10 min to pellet bacterial cells, the supernatant fractions, which contained the phage-displayed scFvs were removed and mixed 1:1 with 1 × PBS containing 4% (w/v) casein and 0.2% (v/v) Tween-20 prior to undergoing ELISA testing as described for the polyclonal phage ELISA above.

### Monoclonal Soluble scFv ELISA

The monoclonal soluble scFv ELISA has been described by van Wyngaardt et al. (36) and Opperman et al. (25). The method is similar to the monoclonal phage ELISA described above, except, instead of rescuing phages with the M13K07 helper phage, soluble scFvs were induced by adding 2 × TY containing 100 μg/ml ampicillin and 3 mM IPTG. The anti-c-Myc MAb 9E10, expressed from the murine hybridoma Mycl-9E10 (Sigma-Aldrich) and the polyclonal rabbit anti-mouse IgG conjugated to horseradish peroxidase (P0260; Dako) detected the secreted soluble scFvs. The ELISA colormetric reaction was performed as described above.

### DNA Sequencing and Sequence Analysis of Phage-Displayed scFvs

Phagemid DNA for monoclonal scFv ELISA positive clones were sequenced [ABI PRISM™ Big Dye™ Terminator Cycling Ready Reaction Kit v.3.0 (Applied Biosystems)]. The clones were inoculated into 2 x TY medium containing 100 μg/ml ampicillin and 20% (w/v) glucose and phagemid DNA was isolated with a QIAprep^®^ Spin Miniprep Kit (Qiagen) as per the manufacturer's instructions. The OP52 forward primer (5′-CCCTCATAGTTAGCGTAACG-3′) and M13 reverse primer (5′-CAGGAAACAGCTATGAC-3′), as well as the ABI PRISM™ Big Dye™ Terminator Cycling Ready Reaction Kit v.3.0 (Applied Biosystems) was used to sequence the single clones (36). An ABI 3100 automated sequencer resolved the extension products and all sequences were edited, assembled and translated using BioEdit v.7.0.9 (45) and Sequencher v5.4.6 (Gene Codes Corporation, Ann Arbor, MI, USA) software.

### Large Scale Expression and Purification of Soluble scFvs

Glycerol stocks (150 μl) of selected phage clones were inoculated in 90 ml of 2 × TY medium (16 g/L tryptone, 10 g/L yeast extract, 5 g/L NaCl) containing 100 μg/ml ampicillin and 2% (w/v) glucose and incubated overnight at 30 °C with shaking. A 1:10 dilution of the overnight culture was prepared in 800 ml of fresh 2 × TY medium containing 100 μg/ml ampicillin and 2% (w/v) glucose and incubation, with shaking, continued for a further 8 h after which the bacterial cells were pelleted at 4,000 × g for 30 min. All traces of glucose-containing 2 × TY media was removed and the bacterial pellet resuspended in 1 L of 2 × TY media containing 100 μg/ml ampicillin and 1 mM IPTG and incubated overnight at 30°C with shaking. The expressed soluble scFv was harvested by pelleting the bacterial cells at 4,000 × g for 30 min.

The bacterial pellets were resuspended in 50 ml of TSA buffer (0.05 M Tris, 0.1 M NaCl, 0.02% NaN_3_, 0.02% sodium azide; pH 8.0) and treated with 0.01% (v/v) of 100 mg/ml lysozyme for 30 min at 30°C. Freshly prepared 200 mM phenylmethylsulfonyl fluoride [0.01% (v/v)] in isopropanol was added to the bacterial suspension and the suspension mixed by inverting. The bacterial suspension was sonicated for 3 min (30 s pulses with 30 s pauses), where after, the bacterial pellet was collected by centrifugation at 15,000 × g for 30 min and the clear lysate filtered through 0.8 and 0.45 μm filters, respectively. The anti-*myc*-Sepharose column coupled with 143 mg of 9E10 Mab (prepared by Janine Frischmuth, the National Bioproducts Institute, Biotechnology division, Pinetown, South Africa) was washed with TSA buffer before the clear lysate (containing soluble scFvs) was loaded through the column via a peristaltic pump. The column was washed with TSA buffer until the spectrophotometer reading at absorbance A_280nm_ fell below 0.3. Soluble scFvs were eluted from the column with elution buffer (0.1 M glycine, 0.14 M NaCl, pH 2.2) and fractions collected. Peak fractions were pooled and the scFvs dialyzed (Sigma-Aldrich, dialysis tubing cellulose membrane, 10 mm flat width), for 48 h at 4°C, in 2 L PBS pH 7.4.

### Binding Specificity of Soluble scFvs

The specificity of the soluble scFvs was tested with an ELISA essentially performed as described for the monoclonal phage ELISA. ELISA plates were coated in duplicate with 30 μg/ml of purified SAT1/KNP/196/91, SAT2/ZIM/7/83, SAT3/KNP/10/90, A22, or A24 viruses as well as with BHK-21 cell extract, 2% (w/v) sucrose and 1 × PBS containing 2% (w/v) milk powder as negative controls.

### Neutralization Assays and Generation of Virus Escape Mutants

IB-RS-2 cells were used to determine the 50% tissue culture infective dose (TCID_50_) of A22 (OIE Terrestrial Manual, 2018). Similarly, BHK-21 cells were used to determine the virus titers for SAT1/KNP/196/91 and SAT3/KNP/10/90. The resulting virus titers were used to calculate the dilutions subsequently used in the virus neutralization test (VNT).

Virus dilutions containing ~500, 50, and 5 infectious particles were prepared in the appropriate cell medium (RPMI for IB-RS-2 cells and GMEM for BHK-21 cells) and were applied in triplicate wells across a microtiter plate and diluted two-fold down the plate. Virus was incubated for 1 h at 37 °C in an atmosphere of 5% CO_2_ after purified scFvs at a concentration between 0.03 and 0.23 mg/ml were added neat to appropriate wells. A control plate without soluble scFvs was included. BHK-21 and IB-RS-2 cells supplemented with 1% (v/v) FCS and antibiotics (virus growth medium, VGM), with a cell count of 0.3 × 10^6^ cells/ml for both cell lines, were subsequently added to the respective microtiter plates. Incubation of microtiter plates then occurred for 72 h at 37°C and fixation and staining with a methylene blue-formaldehyde stain to allow for inspection of the cytopathic effect, which was scored as a measure of neutralization.

To generate virus neutralization escape mutants, the viruses (SAT1/KNP/196/91, SAT3/KNP/10/90, and A22) were passaged under scFv pressure as described by Crowther et al. (19) and Opperman et al. (25). Equal volumes of ca. 25 infectious virus particles were diluted two-fold in GMEM or RPMI medium on a microtiter plate before being mixed with an equal volume of the respective purified scFv (neat), followed by 30 min incubation at 37°C. The virus-scFv complexes were added to either BHK-21 or IB-RS-2 monolayer cells and incubated for 1 h at 37°C. Following incubation, the virus-scFv complexes were removed. Monolayers were washed twice with GMEM or RPMI (Sigma-Aldrich) medium before VGM containing a 1:50 dilution of purified scFvs at a concentration between 0.6 and 4.6 μg/ml were added. All scFvs were tested. Each virus was subjected to four consecutive passages under scFv pressure.

### Characterization of Virus Escape Mutants

Virus escape mutants were then characterized by sequencing the Leader- P1-2A coding region (ca. 3 kb) of the virus genome. RNA extraction was performed using the QIAmp viral RNA kit (Qiagen). cDNA was synthesized with SuperScript III first strand synthesis kit (Invitrogen), using the genome specific primer WDA 5′-GAAGGGCCCAGGGTTGGACTC-3′.

The Leader-P1-2A coding region of the escape viruses were amplified using the Expand Long Template PCR System (Roche) and forward primer NCR1 5′-TACCAAGCGACACTCGGGATCT-3′ and reverse primer WDA 5′-GAAGGGCCCAGGGTTGGACTC-3′. Briefly, each 50 μl PCR reaction mixture consisted of 3 μl of the first strand cDNA reaction mixture, 0.3 μM of each oligonucleotide, 2.5 U of Expand Long Template DNA polymerase, 1 × Expand buffer, 0.75 mM MgCl_2_ and 2 μM of each dNTP. Using a thermocycler (GeneAmp 9700, Applied Biosystems) after initial denaturation at 94°C of 2 min, the reactions were subjected to 35 cycles of 94°C for 20 s, 55°C for 20 s and 68°C for 4 min with a final cycle of 68°C for 7 min to complete the synthesis of all strands.

To determine the nucleotide sequence of the gel-purified amplicons, 0.16 μM of the appropriate oligonucleotide (Table 2) and the ABI PRISM Big Dye Terminator Cycling Ready Reaction kit v3.0 (Applied Biosystems) was utilized. The extension products were resolved on an ABI PRISM™ 3100 automated sequencer (Applied Biosystems) and sequences analyzed using the BioEdit v.7.0.9 (45) and Sequencher v5.4.6 (Gene Codes Corporation, Ann Arbor, MI, USA) software of the ca. 2.2-kb P1-coding region.

**Table 2:** Oligonucleotides used for sequencing the virus escape mutants.

**Oligonucleotide**	^*^Sequence
L internal	5′-GWTACGTCGATGARCC-3′
NCR1	5′-TACCAAGCGACACTCGGGATCT-3′
WDA	5′-GAAGGGCCCAGGGTTGGACTC-3′
SEQ 16	5′-GTGGAACAAGCAGAGAGG T-3′
SEQ 18	5′-CAACTGCAACGTCCTTCTC-3′

* *In selected oligonucleotides, the abbreviation represents ambiguities i.e., W=A or T, R=A or G*.

### Investigation of the SAT1 and SAT3 Soluble scFvs as Capturing Antibodies in an Indirect ELISA

The SAT1 and SAT3 soluble scFvs were tested in an indirect ELISA against a panel of viruses (Table 1) to determine whether it can be used as capturing antibodies in routine testing of suspected FMDV cases. The PEG concentrated viruses were titrated in a liquid phase blocking ELISA [LPBE; (46)] to determine the optimal dilution where an absorbance value at 450 nm (A_450nm_) of ca. one was obtained. The virus dilution of 1:8 was chosen for the scFv ELISA as this was the highest dilution where an A_450nm_ ~1 (with standard deviation of 0.25, observed after the ELISA colorimetric reaction) was obtained for the viruses tested. The purified, neat scFvs (SAT1scFv1 0.03 mg/ml, SAT3scFv1 0.039 mg/ml, and SAT3scFv2 0.09 mg/ml) were used to coat 96-well Maxisorp immunoplates (Nunc^®^) overnight at 4 °C, following which, ELISA plates were washed four times with wash buffer [1 × PBS containing 0.05% (v/v) Tween 20]. As a blocking reagent and negative control, 2% milk powder in 1 × PBS was used. Diluted PEG concentrated virus (1:8) was added to the scFv coated plates and incubated for 1 h at 37°C. Following incubation and washing, serotype specific guinea pig antiserum (typing/detecting antibody, ARC-OVR-VDD) was added (working dilution for SAT1 and SAT2 was 1:100 and SAT3 whilst for A it was 1:50) and plates were again incubated for 1 h at 37°C and then washed. The conjugate (rabbit anti-guinea pig IgG conjugated to horseradish peroxidase, Sigma-Aldrich) diluted at 1:80, was added to respective microtiter plate wells, followed by 1 h at 37°C incubation and washing. The ELISA colorimetric reaction followed using substrate-chromogen solution, consisting of 4 mM 3,3′,5,5′-Tetramethylbenzidine (Sigma-Aldrich) in substrate buffer (0.1 M citric acid monohydrate, 0.1M, disodium hydrogen phosphate; pH 4.5) 0.015% (v/v) H_2_O_2._ Following 10 min incubation at room temperature, the colorimetric reaction was stopped with 1.25M H_2_SO_4_. The A_450nm_ was determined using a Labsystems Multiskan Plus photometer (Thermo Fisher Scientific). The samples were tested in duplicate wells and the absorbance calculated as an average of the two values for each sample. A positive ELISA result was calculated as two-fold the A_450nm_ value of the average negative control.

### Investigation of the SAT1 and SAT3 Soluble scFvs as Detecting Antibodies in an Indirect ELISA

The SAT1- and SAT3-specific soluble scFvs from this study were further investigated for their suitability as a detecting antibody for the FMDV antigen in an ELISA format. 96-well ELISA plates (Nunc^®^) were coated with either SAT1, SAT2, SAT3, or A specific rabbit antiserum. A 1:8 dilution of PEG concentrated viruses (Table 1) were added to the coated 96-well Maxisorp immunoplates (Nunc^®^) and incubated for 1 h at 37°C. Following a wash step with wash buffer i.e., 1 × PBS containing 0.05% (v/v) Tween 20, undiluted scFvs (SAT1scFv1 0.03 mg/ml, SAT3scFv1 0.039 mg/ml, and SAT3scFv2 0.09 mg/ml) were added and ELISA plates incubated for 1 h at 37°C. Microtiter plates were washed and the soluble scFvs that bound to the FMD antigen were detected with the anti-c-Myc antibody clone 9E10 (Sigma-Aldrich) and 1: 1,000 dilution of horseradish peroxidase (HRP)-conjugated polyclonal rabbit anti-mouse IgG (PO260; Dako). The negative control contained 2% milk powder (Elite) in 1 × PBS instead of the scFvs. The substrate/chromogen solution and A_450nm_ determination was performed as described above and a positive A_450nm_ value was considered greater-than or equal to two-fold the average negative control value. The test samples were tested in duplicate and the absorbance calculated as an average of the two values for each test sample. The LPBE was adapted and essentially carried out in conjunction as a comparison of performance of the scFv detecting ELISA and was undertaken as described in the Office International des Epizooties (46).

## Results

### Selection and Identification of Phage-Displayed scFvs Against FMDV SAT1/KNP/196/91, SAT3/KNP/10/90, and A22

**Figure 1:**
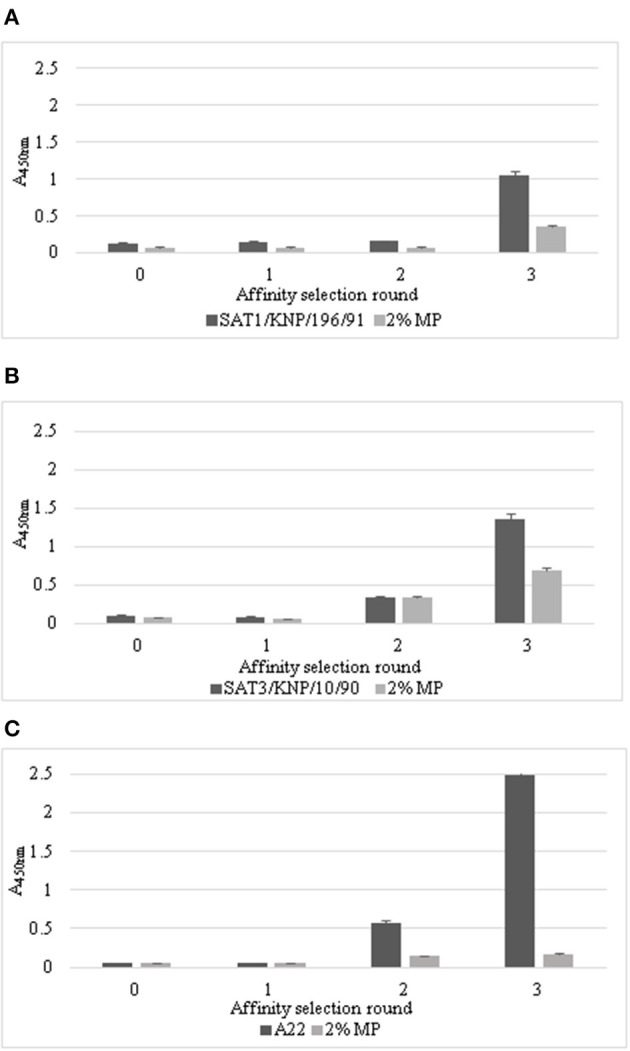
Enrichment of phage-displayed scFvs for **(A)** SAT1/KNP/196/91, **(B)** SAT3/KNP/10/90, and **(C)** A22 using a polyclonal ELISA. Phage-displayed scFvs that bound to SAT1/KNP/196/91, SAT3/KNP/10/90, and A22 were eluted and enrichment of virus specific phage-displayed scFvs (black bars) was determined by a polyclonal phage ELISA of the outputs for three consecutive biopannings. The unpanned aliquot of the *Nkuku*^®^ phage display library was a non-enriched control (selection round 0). The negative control used was 2% milk powder (labeled 2% MP, gray bars). The data are averages ±SD of three repeats.

The large semi-synthetic naïve *Nkuku*^®^ phage display library based on chicken immunoglobulin genes, was panned by exposing the recombinant antibody repertoire to SDG purified virions of the FMD SAT1/KNP/196/91, SAT3/KNP/10/90, and A22 viruses. After three consecutive biopanning rounds, polyclonal phage displayed scFvs were tested in a polyclonal ELISA to evaluate enrichment (Figure 1) for each of the three biopannings to the specific viruses. The phage outputs from the three consecutive selection rounds were tested and an aliquot of the library prior to panning was included as a non-enriched control (Figure 1). Output phages from selection round three resulted in A_450nm_ of 1.05, 1.35, and 2.48 for SAT1/KNP/196/91, SAT3/KNP/10/90, and A22, respectively (Figures 1A–C). The *Nkuku*^®^ non-enriched control (selection round 0) produced A_450nm_ results of 0.13, 0.10, and 0.06 for SAT1/KNP/196/91, SAT3/KNP/10/90, and A22, respectively. An increase of at least eight-fold in the absorbance values after three pannings compared to the absorbance of a pre-panning aliquot of the *Nkuku*^®^ library proved enrichment for phage displayed antibodies for all three FMD viruses (Figures 1A–C).

Phage displayed scFvs specific for FMDV were identified after a helper phage rescue of single bacterial colonies from the third round of panning and tested in ELISA. Both the phage displayed and soluble scFv formats were tested and the results are summarized in Table 3. A total of 94 clones each for SAT1/KNP/196/91 and SAT3/KNP/10/90 and 188 clones for A22 were screened. Twenty clones expressed phage-displayed scFvs specific to SAT1/KNP/196/91 with ELISA signals more than two-fold greater than that of the negative control. Furthermore, of these, seven clones secreted soluble scFvs that bound to SAT1/KNP/196/91. Sequencing of the seven clones revealed one unique binder for SAT1/KNP/196/91, designated SAT1scFv1 (Table 4), which had seven identical clones. Analysis of the 94 clones for SAT3/KNP/10/90 revealed three clones expressing phage-displayed scFvs specific to SAT3/KNP/10/90, all of which secreted soluble scFvs that bound to SAT3/KNP/10/90. Sequencing of these clones indicated two unique binders designated SAT3scFv1 and SAT3scFv2 (Table 4). In addition, of the 188 clones for A22, 25 clones expressed phage-displayed scFvs specific to A22, whilst 9 clones secreted soluble scFvs that bound to A22 and sequencing revealed nine unique binders for A22 designated A22scFv1 to A22scFv9 (Table 4). Interestingly, sequencing results revealed that SAT3scFv2 and A22scFv6 had an identical sequence in all three of the CDR's for the heavy and light chains and are essentially the same binder (Table 4). However, it must be noted that these biopannings were executed independently of each other and at different times and thus the possibility of cross-contamination is ruled out. The result inferred that the SAT3scFv2 and A22scFv6 binders recognize a conserved amino acid (aa) motif on both SAT3/KNP/10/90 and A22 viruses. The soluble scFvs were subsequently successfully purified by means of affinity chromatography and further characterized.

**Table 3:** Screening for scFvs following three rounds of biopanning.

**FMD serotype**	**Number of clones tested**	**Phage binders**	**Soluble scFv binders**	**Unique sequences/binders**
SAT1/KNP/196/91	94	20	7	1
SAT3/KNP/10/90	94	3	3	2
A22	188	25	9	9

**Table 4:** Amino acid sequence alignment of the complementary determining regions (CDR) of the heavy and light chains of the SAT1/KNP/196/91, SAT3/KNP/10/90, and A22-specific soluble scFvs panned from the *Nkuku*^®^ library.

**scFv**	**Heavy Chain**			**Light chain**		
	**Complementary determining region**			**Complementary determining region**		
	**CDR1**	**CDR2**	**CDR3**	**CDR1**	**CDR2**	**CDR3**
SAT1scFv1	SSHGMF	EITN−−TGSYAAYGAAV	CAKSSYECTSSCWGNTGWID	SGDSSG−−−−YGYG	YNNNKRPS	GTED−GITDAGI
SAT3scFv1	SSNGMA	AISSRD−GSGTGYGSAV	CAKPVKGMY−−−−−−−−−ID	SGGTYYA−−−−−−−	YDNTNRPS	GAYDSS−TYAGI
SAT3scFv2	SSFNMG	AINND−−GGGTAYGSAV	CAKSVDDSWNV−−−−−DSID	SGGGSYAGS−YYYG	YDNTKRPS	GSYDSS−−−GGI
A22scFv1	SSYSMQ	GIGS−−DGSDTAYGAAV	CTKCGYGGS−GYCWYAGDID	SGGGNE−−−−−−YG	YWNDKRPS	GSYDSSA−−−GI
A22scFv2	SSYEMQ	GIEN−−DGSNPNYGAAV	CAKSAYGGSWGGYIPTDSID	SGG−SSS−−−−YYG	YDNTNRPS	GSFDSSTTV−GI
A22scFv3	SDYAMG	GIGTSADGSSTAYGAAV	CTRTGAAE−−−−−−−−−DID	SGG−SSS−−−−YYG	YANTNRPS	GSSDSTY−−VGI
A22scFv4	SSHGMG	SISR−−DSSYTDYGPAV	CTKSAGPYVNGDN−−−−−ID	SGGGRYAGNYYYYG	YSNNQRPS	GSADSNSTDGVT
A22scFv5	SDYGMS	EITND−−DSWTGYGAAV	CAKNDYYSLF−−−−−−−−ID	SG−−DSN−−YYGYS	YDNDKRPS	GSADSSA−−−VI
A22scFv6	SSFNMG	AINND−−GGGTAYGSAV	CAKSVDDSWNV−−−−−DSID	SGGGSYAGS−YYYG	YDNTKRPS	GSYDSS−−−GGI
A22scFv7	SSYGMG	GIEN−−DGRYTGYGSAV	CAKDIYG−VGGGAFGADTID	SGG−SYS−−−−−YG	YDNTNRPS	GSIDSSY−V−GI
A22scFv8	SSYSMQ	GIGS−−DGSDTAYGAAV	CTKCGYGGS−GYCWYAGDID	SGGGS−−−−−−YYG	YSNNQRPS	GSYDNSA−−−GI
A22scFv9	SSYPMG	AISN−−DGSYTGYGAAV	CAKDAYSYTTTGGWYVDEID	SGGGS−−−−−−YYG	YDNTNRPS	GGIDSTD−−−AA

### Binding Specificity of Soluble scFvs to FMDV

**Figure 2:**
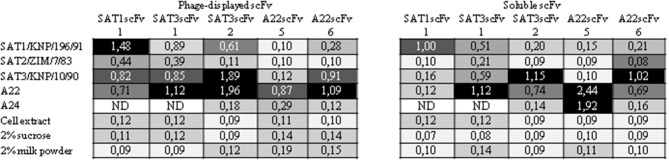
Heat map representing color-coded ELISA A_450nm_ absorbance of the indirect ELISA testing the specificity of scFvs i.e., SAT1scFv1, SAT3scFv1, SAT3scFv2, A22scFv5, and A22scFv6. The remainder of the A22 scFv phage and soluble binders only reacted to A22 with no significant reaction to any of the other viruses or reagents (data not shown). Both the phage-displayed and soluble scFvs were tested against FMDV A22, A24, SAT1/KNP/196/91, SAT2/ZIM/7/83, and SAT3/KNP/10/90 SDG purified viruses as well as BHK-21 cell extract, 2% sucrose, and 2% milk powder (MP). An ELISA signal two-fold that of the 2% milk powder soluble scFv absorbance was considered a positive result.

The specificity of the virus-specific soluble scFvs were determined by measuring their ability to bind to purified, complete 146S virions of viruses from serotypes A, SAT1, SAT2, and SAT3 in an ELISA. Negative controls were BHK-21 cell extract, 2% sucrose, and 2% milk powder (Figure 2). SAT1scFv1 phage-displayed binder cross-reacted with A22, SAT2/ZIM/7/83, and SAT3/KNP/10/90, however, the soluble scFv format did not exhibit any cross-reactivity (Figure 2). SAT3scFv1 phage-displayed and soluble scFv cross-reacted with A22 and SAT1/KNP/196/91 whilst there was borderline cross-reactivity with SAT2/ZIM/7/83 observed for the phage-displayed scFv (Figure 2). The amino acid sequence of the heavy and light chain complementary determining regions (CDRs) of SAT3scFv2 and A22scFv6 are identical and the cross-reactivity between SAT3scFv2 and A22 as well as A22scFv6 and SAT3/KNP/10/90 was observed with both the phage-displayed and soluble scFv (Figure 2). SAT3scFv2 phage-displayed scFv also cross-reacted with SAT1/KNP/196/91, whereas the soluble scFv did not cross-react (Figure 2). A22scFv5 soluble scFv showed cross-reactivity to A24 virus and for the phage-displayed scFv, borderline cross-reactivity was observed (Figure 2). There was no cross-reactivity observed with the A22scFv1, A22scFv2, A22scFv3, A22scFv4, A22scFv7, A22scFv8, and A22scFv9 binders with SAT1/KNP/196/91, SAT2/ZIM/7/83, SAT3/KNP/10/90, or A24 viruses. No cross-reactivity was observed with the reagents used for virus propagation and purification. The specificity investigations of the scFvs showed that the phage-displayed scFvs exhibited more prominent cross-reactivity when compared to the soluble scFvs. Due to this, further investigations for this study was continued with the soluble scFvs.

### Neutralization and Escape Mutant Investigations of the Identified scFvs

The ability of the soluble scFv's against SAT1, SAT3, and A22 to neutralize the SAT1/KNP/196/91, SAT3/KNP/10/90, or A22 viruses, respectively, *in vitro* was investigated. The SAT1scFv1 was unable to neutralize FMDV SAT1/KNP/196/91. Similarly, SAT3scFv1 and SAT3scFv2 binders were unable to neutralize the SAT3/KNP/10/90 virus. Additionally, A22scFv6, being essentially the same binder as SAT3scFv2, was unable to neutralize A22 virus *in vitro*. Nonetheless, three of the nine A22 soluble scFv binders, i.e., A22scFv1 (0.16 mg/ml), A22scFv2 (0.23 mg/ml), and A22scFv8 (0.18 mg/ml), were able to neutralize A22 *in vitro*. The neutralization titers (TCID_50_) are indicated in Table 5.

**Table 5:** The 50% tissue culture infectious dose (TCID_50_) of A22 when neutralized by the A22 scFvs.

scFv^*^	**Neutralization titer (TCID_**50**_/50 μl)**
A22scFv1	0.5
A22scFv2	0.63
A22scFv8	0.63

* *Titer of A22 (without scFvs) was 1.25 TCID_50_/50 μl*.

**Figure 3:**
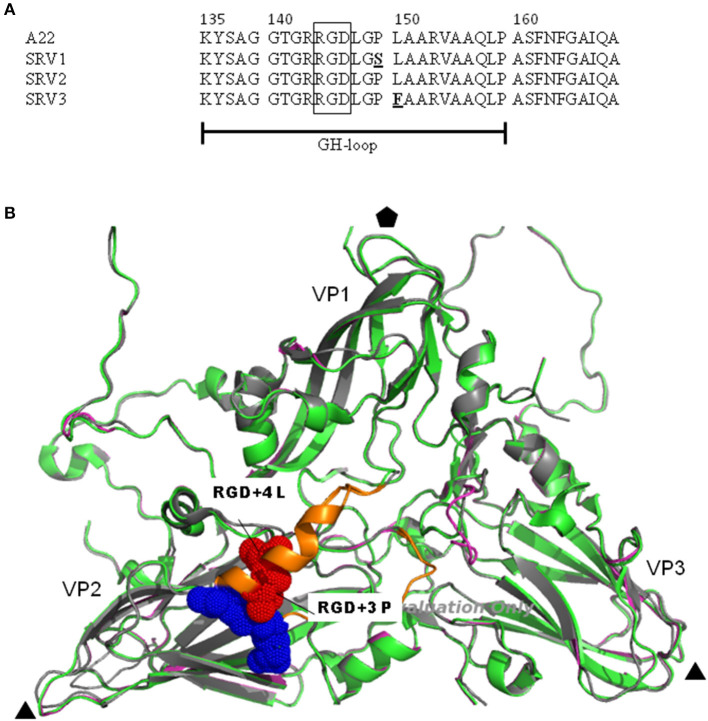
**(A)** The A22 virus escape mutant substitutions are indicated in an aa alignment of the VP1 GH-loop of A22. The proline to serine (Pro149➔Ser) substitution at position 149 of VP1 for SRV1 and a leucine to a phenylalanine (Leu150➔Phe) substitution at position 150 of VP1 for SRV3 is shown in bold and underlined. The RGD motif is blocked. **(B)** The substitutions are shown as red dots on a cartoon model of the FMDV capsid proteins in a crystallographic protomer. The inferred 3-D structural model was rendered by Pymol v 1.8 (DeLano Scientific LLC). The VP1 GH-loop is absent from the A22 complete (PDB: 4GH4) and empty capsid (PDB: 5D8A) structures due to an instable loop conformation. However, the serotype O capsid structure (PDB: 1FOD) was reported with a VP1 GH-loop (47). The serotype A complete and empty capsid protomers (colored in green and magenta) were superimposed on the O protomer (in gray), showing the position of the VP1 GH-loop in orange and the receptor-binding RGD sequence as blue dots. The three-fold axis is depicted by the black triangles and the five-fold axis of the capsid by the black pentagon. The positions of the outer-capsid proteins, VP1, VP2, and VP3 are indicated.

FMDV A22 was serially passaged in the presence of soluble A22scFv1, A22scFv2, and A22scFv8 to select viruses from the A22 quasispecies population that escape neutralization by the soluble scFvs. Thus, the A22 viruses that escaped neutralization by soluble scFvs A22scFv1, A22scFv2, and A22scFv8 were designated scFv resistant virus (SRV) 1, 2, and 3, respectively. Following four consecutive passages under scFv pressure, the P1 nucleotide, and aa sequences were determined for SRV1, SRV2, and SRV3. Comparative analysis of the aa sequences of the SRVs compared to the parental A22 sequence indicated that SRV1 exhibited one aa substitution i.e., from a non-polar proline (Pro) to a polar serine (Ser) change at VP1 aa position 149 i.e., RGD+3 (Pro149➔Ser) (Figure 3). Interestingly SRV2 had no aa changes occurring in the P1 region, but SRV3 exhibited a single aa substitution of a leucine (Leu) to a phenylalanine (Phe) at position 150 of VP1 i.e., RGD+4 (Leu150➔Phe) (Figure 3). The aa substitutions for SRV1 and SRV3 occurred in the surface exposed and structurally flexible VP1 βG-βH loop, downstream of the RGD sequence.

The A22 virus was neutralized by three soluble scFvs and SRVs for A22, which showed a potential binding site for two of the scFvs in the GH-loop of the VP1 protein. The three CDRs of the H-chain of A22scFv1 and A22scFv8 are identical while the L-chain of A22scFv1 and A22scFv8 displayed different sequences (Table 4). Soluble scFvs for SAT1/KNP/196/91 and SAT3/KNP/10/90 viruses did not neutralize the respective viruses. Thus, the SAT1 and SAT3 scFvs were investigated for its potential use as diagnostic reagents in an ELISA.

### SAT Virus-Specific scFvs as a FMDV Capturing Antibody in a Sandwich ELISA

To determine whether the SAT1 and SAT3 soluble scFv's retain the correct conformation to act as capturing reagents in a diagnostic sandwich ELISA, the soluble scFvs were coated directly onto maxisorp immunoplates. The antigen-binding activity of the immobilized soluble SATscFv1 was tested against a panel of PEG concentrated SAT1 (*n* = 11) viruses whilst soluble SAT3scFv1 and SAT3scFv2 was tested against a panel of SAT3 (*n* = 12) viruses (Table 1).

**Figure 4:**
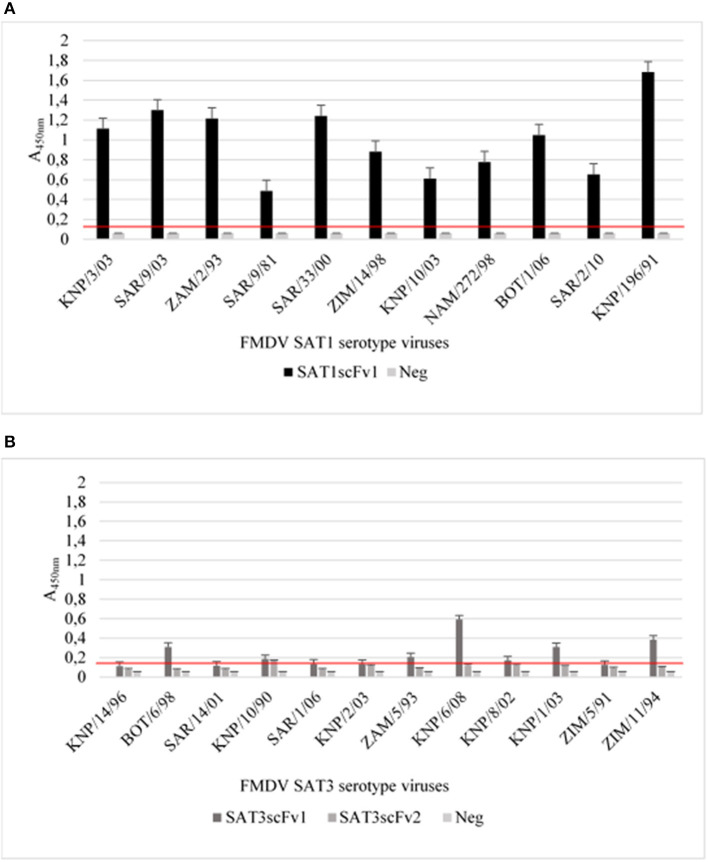
A sandwich ELISA with soluble scFvs as capturing antibodies. The SAT1scFv1 was tested with a panel of PEG concentrated SAT1 viruses **(A)** and SAT3scFv1 and SAT3scFv2 was tested with the SAT3 viruses **(B)**. For the negative control (neg), 2% milk powder was included in the assays replacing the soluble scFvs coating the plate. The data are means ± SD of two independent experiments. An ELISA signal more than two-fold that of the negative control A_450nm_ was considered a positive result and the cut-off is indicated by a red line.

The results revealed that soluble SAT1scFv1 successfully captured the panel of SAT1 viruses tested as ELISA signals of A_450nm_ ≥0.48 and ≤ 1.68 were obtained (Figure 4A). Weak positive A_450nm_ of >0.4, <0.9 was observed when SAT1scFv1 captured SAT1/SAR/9/81, SAT1/ZIM/14/98, SAT1/KNP/10/03, SAT/NAM/272/98, and SAT1/SAR/2/10 (Figure 4A). Additionally, strong positive signals A_450nm_ >1, <1.68 for SAT1/KNP/3/03, SAT1/SAR/9/03, SAT1/ZAM/2/93, SAT1/SAR/33/00, SAT1/BOT/1/06, and SAT1/KNP/196/91 were observed when SAT1scFv1 was the capturing reagent (Figure 4A).

SAT3scFv1 and SAT3scFv2 soluble scFvs did not react efficiently with the SAT3 viruses when applied as a capturing reagent and displayed A_450nm_ signals not significantly higher than the negative controls. Weak positive results were obtained for SAT3scFv1 with SAT3/KNP/6/08 (A_450nm_ 0.6) (Figure 4B). SAT3scFv1 showed similar reactivity to SAT3/BOT/6/98 (A_450nm_ 0.31), SAT3/KNP/1/03 (A_450nm_ 0.31), and SAT3/ZIM/11/94 (A_450nm_ 0.40) viruses indicating this scFv may recognize the same epitope on the virion for these viruses (Figure 4B). Additionally, absorbance values of both the SAT3 scFvs in the capturing ELISA against the virus used for the panning i.e., SAT3/KNP/10/90 was <0.2.

Overall, the ELISA results using the scFvs as capturing reagents indicate that the soluble SAT1scFv1 was able to successfully bind to the polystyrene ELISA plate and react to viruses within the SAT1 serotype. The SAT3scFv1 and SAT3scFv2 exhibited no or borderline reactivity with the SAT3 viruses tested. The low signals may be attributed to the conformational changes of the scFvs when binding to the ELISA plate. Furthermore, aa differences in the viral proteins may result in the variable ELISA signals observed.

### SAT Virus-Specific scFvs as a FMDV Detecting Antibody in an ELISA

The soluble scFvs, SAT1scFv1, SAT3scFv1, and SAT3scFv2, were also applied as detecting antibodies in a sandwich ELISA. FMD virus was captured by polyclonal rabbit antiserum and the soluble scFvs was used to detect the 146S virus particles using the panel in Table 1. The standard diagnostic sandwich ELISA used for antigen detection (46) was performed concurrently as a comparison of the scFv ELISA performance.

**Figure 5:**
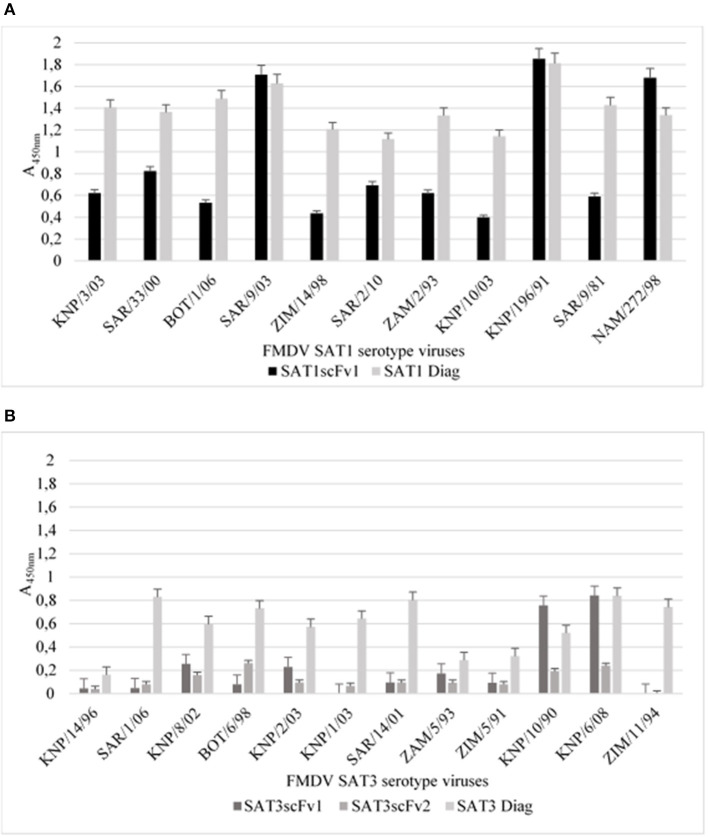
Sandwich ELISA using soluble SAT1scFv1, SAT3scFv1, and SAT3scFv2 as detecting reagents. The standard diagnostic sandwich ELISA used for FMD antigen detection (46) [used as a positive control (SAT1 diag or SAT3 diag)] was adapted, where the detection antibody was replaced with a soluble SAT scFv. The ELISA was executed to determine the detecting potential of soluble SAT1scFv1 to PEG concentrated SAT1 viruses **(A)** and for SAT3scFv1 and SAT3scFv2 to PEG concentrated SAT3 serotype viruses **(B)**. For the negative control, 2% milk powder was included in the assays replacing the virus component. The negative control ELISA background signal was deducted when plotting the ELISA A_450nm_ result. The data are means ± SD of two independent experiments.

Results showed that the diagnostic antigen detection ELISA was able to detect all viruses tested and produced positive ELISA signals (Figures 5A,B). The ELISA assay using SAT1scFv1 as a detecting antibody revealed two characteristic reactivity profiles against the panel of SAT1 viruses, i.e., (i) A_450nm_ >1.4 was observed for SAT1/SAR/9/03, SAT1/KNP/196/91, and SAT1/NAM/272/98 viruses and (ii) A_450nm_ absorbance values of ≥0.4, ≤ 0.82 for the remaining eight SAT1 viruses in the panel (Figure 5A).

The soluble SAT3scFv1 and SAT3scFv2 showed low absorbance signals for the SAT3 viruses tested (Figure 5B). Absorbance signals (A_450nm_) of 0.76 and 0.84 were observed for SAT3/KNP/10/90 and SAT3/KNP/6/08, respectively when SAT3scFv1 was the detecting antibody in the ELISA. All other A_450nm_ values were below 0.26 for the SAT3 viruses tested with SAT3scFv1 and SAT3scFv2 (Figure 5B).

Taken together, the results indicate that the two SAT3 specific scFvs showed poor potential as a detecting ELISA antibody whilst conversely, SAT1scFv1 showed good potential for incorporation as a detecting antibody in a diagnostic ELISA.

## Discussion

Serotype-specific serological tests for FMD detect antibodies against the structural proteins that are elicited by either vaccination or infection (48). Both the solid-phase competition (SPCE) and LPBE ELISAs for SAT1, SAT2, and SAT3 are OIE recognized and well established assays. Considering the high genetic diversity of the SAT-type viruses (2, 6, 26, 49), there is a continuous need for improvement of these assays as cross-reactivity has been noted with the SAT types using this assay. Additionally, for FMD vaccine matching where the antigenic variability of field virus strains is measured against current vaccine strains, the virus neutralization assay is utilized. This assay, however, is laborious and can cause a delay in decision making regarding FMD control measures. To address this shortcoming, utilizing MAbs or virus-specific scFv's for FMDV that recognize virus exposed antigenic epitopes in an ELISA format where results can be obtained timeously and accurately, was explored. These scFv's or small recombinant MAbs can be produced in large quantities (50) and can also be beneficial in predicting epitopes, which can in turn be used in the design of improved FMD chimeric vaccines containing various antigenic sites that can elicit a wide immunological response or protection in vaccinated animals. To this end, phage display technology was explored.

The *Nkuku*^®^ phage-display library has previously been panned on FMDV to obtain unique scFv binders for serotype SAT2 (25). Opperman et al. (25) has shown that one SAT2-specific soluble scFv neutralized SAT2/ZIM/7/83. This scFv interacts with a novel epitope at residue position 159 of VP1 and was applied in a scFv-based ELISA assay. We broadened this study by including FMDV serotype SAT1, SAT3, and A. Using naïve phage display libraries allows for the selection of recombinant antibodies where unique paratopes bind to exposed and complementary parts of the immobilized antigen. Thus, the possibility of obtaining antigen-specific binders would depend on the presence and accessibility of suitable surface-exposed structures of the antigen, which in this case, was the FMD virion. Another factor to consider when using phage display technology, is that the quality and the size of the naïve library plays an important role in the success of phage display (51), as paratopes that are not present within the library cannot be isolated.

In this study, the biopanning process with SAT1/KNP/196/91, SAT3/KNP/10/90, and A22 viruses resulted in unique FMDV-specific scFv binders i.e., one for SAT1, two for SAT3, and nine for serotype A. The nine A22 binders attained in this study is unique. Although none of the SAT scFv binders showed neutralization capability, three of the nine A22 binders exhibited neutralization. Of the three A22 neutralizing binders, two binders i.e., A22scFv1 and A22scFv8 had the same heavy chain sequences and only differed in the light chain region sequences. This characteristic is of interest as Hamers-Casterman et al. (52) showed that the V_H_ play a more important role in the binding of antibody fragments to antigens than V_L._ In addition, investigations by Williamson and Matthews (53) showed that three neutralizing scFvs against pertussis toxin all had the same heavy chain sequences and were related. Thus, to obtain more neutralizing scFvs, one could modify only the heavy chain of non-neutralizing scFvs of differing V_Ls_ to be the same as their neutralizing counterparts.

For the generation of escape mutants for FMDV serotype A using the neutralizing scFvs, no aa changes were observed in the P1 region for SRV2, which may be because the mutation occurred in the minor population and due to Sanger sequencing, it was not detected. Additionally, the aa substitutions for SRV1 and SRV3 occurred in the surface exposed and structurally flexible VP1 βG-βH loop, downstream of the RGD sequence. The position is identical to the identified FMDV serotype A antigenic site I and furthermore, the βG-βH loop residues 140–160 have been shown to play an important role in antigenicity in most FMDV serotypes (15–17, 19, 54–57). Additionally, changes at the conserved aa leucine residue 150 has also been shown i.e., L➔P or L➔R on escape mutants pressured by using soluble integrins (58, 59). SRV1 and SRV3 alone did not solve the binding footprint of the A22scFv1 and A22scFv8 binders, respectively. It is reasonable to expect different binding footprints on the virion for the two scFvs even though they have a common binding site at VP1 aa position 149/150. For SRV1, there was a Pro to Ser aa change at VP1 position 149 (RGD +3 position) and for SRV3 the aa change occurred at VP1 position 150, RGD+4 i.e., from a Leu to a Phe. The residues succeeding the RGD motif are important for receptor recognition (58, 60) and the RGD is flanked on both sides by hypervariable sequences, which delivers a domain that is capable of adopting different conformations. Both residues are highly conserved in serotype A viruses and the substitution of amino acid residues at position 149 or 150 of MAR-viruses have been described for A_10_, A_12_, and A_24_ (15, 16, 57). Opperman et al. (25) showed with scFv neutralization investigations of FMDV SAT2/ZIM/7/83, an aa change at the base of the GH loop i.e., VP1 position 159 where there was an Arg to His change. Furthermore, a synthetic peptide ELISA confirmed VP1 aa 159 as an important residue in the epitope to which the SAT2 scFv binds (25). These investigations lead us to postulate that the epitope site for the A22scFv1 and A22scFv8 binding involves the VP1 aa position 149 and 150, respectively which is part of the antigenic sequence GDLGSLA for serotype A viruses (15). However, future investigations with SRV1 and SRV3, will be to derive a synthetic peptide from the predicted epitopic site and to confirm results with a synthetic peptide blocking ELISA.

A unique finding from this study is the result of two soluble, non-neutralizing scFvs, each from different FMDV serotypes but having the same heavy and light chain sequences i.e., SAT3scFv2 and A22scFv6. It is postulated that a common epitope between SAT3/KNP/10/90 and A22 resulted in the same soluble scFv from the antibody repertoire of the *Nkuku*^®^ library. Neither of the two scFv's were able to neutralize the respective viruses and thus it was impossible to identify a binding site for the two scFv's on the viral capsid. Competing monoclonal antibody studies or capsid protein peptide libraries will be used to confirm the common or cross-reacting epitope between A22 and SAT3.

From the specificity analysis, the SAT1 and SAT3 soluble scFvs from this study bound to complete 146S virions of the virus used for biopanning i.e., SAT1/KNP/196/91 and SAT3/KNP/10/90, respectively. However, the phage displayed scFv formats did show cross-reactivity across the FMD serotype viruses tested. The SAT1 phage displayed scFv was found to bind to complete 146S virions of SAT2/ZIM/7/83 and SAT3/KNP/10/90 and similarly, the SAT3 phage-displayed scFvs were found to bind SAT1/KNP/196/91 and SAT2/ZIM/7/83 146S virions. Cross-reactivity of scFvs is not uncommon as in a study by Toth et al. (61), which obtained scFv clones against the potato leafroll virus where 7 clones did not cross-react with other luteoviruses whilst 4 clones did. Additionally, Toth et al. (61) proved that the cross-reacting scFvs are directed against continuous epitopes that are present on the coat proteins of certain related luteoviruses whereas the scFvs that did not show cross-reactivity, bound to discontinuous or conformation-dependant epitopes that are specific to potato leafroll virus. A major continuous FMDV epitope is located in the GH loop spanning VP1 residues around positions 140–160 (62, 63). Thus, the cross-reacting phage-displayed SAT1 and SAT3 scFvs from this study may possibly be recognizing continuous epitopes of the SAT serotype viruses and should be investigated further in this regard.

The SAT soluble scFvs, which had reduced cross-reactivity compared to the phage displayed scFvs, were further investigated for their possible use as diagnostic reagents in an ELISA format as a FMDV capturing and a detecting reagent. For both ELISA formats, the one SAT1 soluble scFv was able to produce high reactivity to the various SAT1 PEG concentrated viruses tested implying that there was little or no effect on its paratope when coated on polystyrene plates and was thus a good capturing reagent. However, the two SAT3 scFvs produced A_450nm_ signals just above the positive cut-off absorbance as a FMDV capturing reagent and a detecting reagent, which is not acceptable as a positive result for a diagnostic ELISA. It is vital for a capturing antibody in an ELISA assay to be efficiently immobilized onto the ELISA plate such that it is able to retain both the antibody conformation and the antigen-binding activity (64). Furthermore, important factors such as surface charge, hydrophobicity, co-adsorption of or exchange with surfactants, and other proteins play a role in determining stability and specificity of absorbed antibodies in ELISA assays (65, 66). These factors may have played a role to reduce the performance of the scFv ELISAs in this study. For conventional ELISAs where complete monoclonal or polyclonal antibodies are used, immobilization onto the ELISA plates occur via physical adsorption (66–69). Conversely, immobilization of small antibody fragments such as scFvs onto plastic surfaces causes unfavorable conformational changes to occur (66). Essentially, a hydrophobic interaction occurs in the linked V_H_ and V_L_ regions that forms a paratope resulting in a conformational change of the antigen-binding domain, which in turn results in decreased antigen-binding activity (66, 70, 71).

The analytical specificity of the SAT scFvs for the capturing and detecting SAT1 and SAT3 ELISAs showed that the soluble scFvs were specific for the respective serotype viruses tested and no cross-reactivity was observed. Additionally, the analytical sensitivity of both the capturing and detecting ELISAs for SAT1 and SAT3 scFvs against the SAT1 and SAT3 viruses, respectively, was found to detect all PEG concentrated viruses at a 1:8 dilution tested albeit the low A_450nm_ signals for the SAT3 scFv ELISAs. The SAT1scFv1 shows promise as a good detecting and immunocapture reagent due to the high reactivity for the SAT1 viruses tested. Studies have shown that when scFv fragments are utilized as a soluble protein and are not within the phage display system, low expression levels, or a low inherent affinity can occur (36). Also, the monomeric scFv fragments can have moderate binding affinities when binding to a large multivalent antigen like FMDV, which is in contrast to greater binding affinities that can be achieved by the multivalent display on the phage (72–74). To overcome the low SAT3 scFv ELISA signals, random mutations can be introduced in the gene coding for the scFv and the length of the linker within the scFv, increasing the bacterial expression of the scFvs and thus increasing the ELISA signal (41). Another approach is to stabilize the scFv to other proteins whilst retaining functionality. For example, fusion or linking to other proteins such as constant light chain domain (75), leucine zipper dimerization domain (76), Fc fragment (CH2 and CH3 domains) of mouse IgG1 (77), and alkaline phosphatase (78). Such approaches may be used in future studies to enhance the diagnostic potential of the SAT1 and SAT3 scFvs from this study.

This study has been beneficial to gain unique recombinant antibodies against FMDV SAT1, SAT3, and A serotype viruses. Although the SAT scFvs did not neutralize FMDV, the potential was shown as ELISA reagents, especially for SAT1scFv1. Further investigation and validations of SAT1scFv1 will be continued to improve ELISA absorbance signal.

## Data Availability Statement

The datasets presented in this study can be found in online repositories. The names of the repository/repositories and accession number(s) can be found in the article/supplementary material.

## Author Contributions

MC: majority of the experimental work, troubleshooting, result and data analysis, and manuscript composition and editing. PO: scFv expertise and training, experimental work, troubleshooting, result and data analysis, manuscript editing, and research grant acquisition. LR: serotype A experimental work, troubleshooting, result and data analysis, and manuscript editing. JFe and WW: scFv expertise advisor, Nkuku library, and manuscript editing. JFr: scFv column development, scFv purification training and expertise/advisor, and manuscript editing. ER: research grant acquisition, PI on grant at PIADC, project advisor, and manuscript editing. FM: research grant acquisition, PI on grant at ARC, project advisor, scFv expertise, and manuscript editing. All authors contributed to the article and approved the submitted version.

## Conflict of Interest

The authors declare that the research was conducted in the absence of any commercial or financial relationships that could be construed as a potential conflict of interest.

## Abbreviations

FMD: foot-and-mouth disease
FMDV: FMD virus
scFv: single-chain variable fragment
MAb: monoclonal antibody.

## References

[1] Scott KA Maake L Botha E Theron J Maree FF. Inherent biophysical stability of foot-and-mouth disease SAT1, SAT2 and SAT3 viruses. Virus Res. (2019) 15:45–55. 10.1016/j.virusres.2019.02.012 30807778

[2] Knowles NJ Samuel AR. Molecular epidemiology of foot-and-mouth disease virus. Virus Res. (2003) 91:65–80. 10.1016/S0168-1702(02)00260-5 12527438

[3] Mateu MG Da Silva JL Rocha E De Brum DL Alonso A Enjuanes L. Extensive antigenic heterogeneity of foot-and-mouth disease virus of serotype C. Virology. (1988) 167:113–24. 10.1016/0042-6822(88)90060-8 2460992

[4] Martínez MA Dopazo J Hernández J Mateu MG Sobrino F Domingo E. Evolution of the capsid protein genes of foot-and-mouth disease virus: antigenic variation without accumulation of amino acid substitutions over six decades. J Virol. (1992) 66:3557–65. 10.1128/JVI.66.6.3557-3565.1992 1316467 PMC241137

[5] Domingo E Diez J Martinez MA Hernandez J Holguin A Borrego R. New observations on antigenic diversification of RNA viruses: antigenic variation is not dependent on immune selection. J Gen Virol. (1993) 74 (Pt 10):2039–45. 10.1099/0022-1317-74-10-2039 7691985

[6] Maree FF Blignaut B Aschenbrenner L Burrage T Rieder E. Analysis of SAT1 type foot-and-mouth disease virus capsid proteins: influence of receptor usage on the properties of virus particles. Virus Res. (2011) 155:462–72. 10.1016/j.virusres.2010.12.002 21167231

[7] Paton DJ Sumption KJ Charleston B. Options for control of foot-and-mouth disease: knowledge, capability and policy. Philos Trans R Soc Lond Ser B Biol Sci. (2009) 364:2657–67. 10.1098/rstb.2009.0100 19687036 PMC2865093

[8] Kitching RP Hutber AM Thrusfield MV. A review of foot-and-mouth disease with special consideration for the clinical and epidemiological factors relevant to predictive modelling of the disease. Vet J. (2005) 169:197–209. 10.1016/j.tvjl.2004.06.001 15727911

[9] Longjam N Tayo T Antigenic variation of foot and mouth disease virus - an overview. Vet World. (2011) 4:475–9. 10.5455/vetworld.2011.475-479

[10] McCullough KC De Simone F Brocchi E Capucci L Crowther JR Kihm U. Protective immune response against foot-and-mouth disease. J Virol. (1992) 66:1835–40. 10.1128/JVI.66.4.1835-1840.1992 1312607 PMC288969

[11] Mateu MG Hernández J Martínez MA Feigelstock D Lea S Pérez JJ. Antigenic heterogeneity of a foot-and-mouth disease virus serotype in the field is mediated by very limited sequence variation at several antigenic sites. J Virol. (1994) 68:1407–17. 10.1128/JVI.68.3.1407-1417.1994 8107204 PMC236594

[12] Juleff ND Interactions of foot-and-mouth disease virus with cells in organised lymphoid tissue influence innate and adaptive immune responses (thesis). The University of Edinburgh (2009). Available online at: http://hdl.handle.net/1842/4256

[13] Mahapatra M Hamblin P Paton DJ. Foot-and-mouth disease virus epitope dominance in the antibody response of vaccinated animals. J Gen Virol. (2012) 93 (Pt 3):488–93. 10.1099/vir.0.037952-0 22158876

[14] Taboga O Tami C Carrillo E Núñez JI Rodríguez A Saíz JC. A large-scale evaluation of peptide vaccines against foot-and-mouth disease: lack of solid protection in cattle and isolation of escape mutants. J Virol. (1997) 71:2606–14. 10.1128/JVI.71.4.2606-2614.1997 9060612 PMC191381

[15] Thomas AAM Woortmeijer RJ Puijk W Barteling SJ. Foot-and-mouth disease virus type A10. J Virol. (1988) 62:2782–9. 10.1128/JVI.62.8.2782-2789.1988 2455819 PMC253712

[16] Baxt B Vakharia V Moore DM Franke AJ Morgan DO. Analysis of neutralizing antigenic sites on the surface of type A12 foot-and-mouth disease virus. J Virol. (1989) 63:2143–51. 10.1128/JVI.63.5.2143-2151.1989 2467993 PMC250631

[17] Bolwell C Clarke BE Parry NR Ouldridge EJ Brown F Rowlands DJ. Epitope mapping of foot-and-mouth disease virus with neutralizing monoclonal antibodies. J Gen Virol. (1989) 70 (Pt 1): 59–68. 10.1099/0022-1317-70-1-59 2471783

[18] Kitson JDA McCahon D Belsham GJ. Sequence analysis of monoclonal antibody resistant mutants of type O foot and mouth disease virus: evidence for the involvement of the three surface exposed capsid proteins in four antigenic sites. Virology. (1990) 179:26–34. 10.1016/0042-6822(90)90269-W 1699353

[19] Crowther JR Rowe CA Butcher R. Characterization of monoclonal antibodies against a type SAT 2 foot-and-mouth disease virus. Epidemiol Infect. (1993) 111:391–406. 10.1017/S0950268800057083 7691630 PMC2271387

[20] Asfor AS Upadhyaya S Knowles NJ King DP Paton DJ Mahapatra M. Novel antibody binding determinants on the capsid surface of serotype O foot-and-mouth disease virus. J Gen Virol. (2014) 95 (Pt 5):1104–16. 10.1099/vir.0.060939-0 24584474 PMC3983758

[21] Mahapatra Mana Yuvaraj S Madhanmohan M Subramaniam S Pattnaik B Paton DJ Srinivasan VA. Antigenic and genetic comparison of foot-and-mouth disease virus serotype O Indian vaccine strain, O/IND/R2/75 against currently circulating viruses. Vaccine. (2015) 33:693–700. 10.1016/j.vaccine.2014.11.058 25500306 PMC4315132

[22] Mateu MG Martínez MA Capucci L Andreu D Giralt E Sobrino F. A single amino acid substitution affects multiple overlapping epitopes in the major antigenic site of foot-and-mouth disease virus of serotype C. J Gen Virol. (1990) 71 (Pt 3):629–37. 10.1099/0022-1317-71-3-629 1690261

[23] Sanyal A Venkataramanan R Pattnaik B. Antigenic features of foot-and-mouth disease virus serotype Asia1 as revealed by monoclonal antibodies and neutralization-escape mutants. Virus Res. (1997) 50:107–17. 10.1016/S0168-1702(97)00058-0 9282776

[24] Grazioli S Moretti M Barbieri I Crosatti M Brocchi E Use of Monoclonal Antibodies to Identify and Map New Antigenic Determinants Involed in Neutralization of FMD Viruses Type SAT 1 and SAT 2. Paphos: European Commission for the Control of Foot-and-Mouth Disease: International Control of Foot-and-Mouth Disease: Tools, Trends and Perspectives (2006). p. 287–297.

[25] Opperman PA Maree FF Van Wyngaardt W Vosloo W Theron J. Mapping of antigenic determinants on a SAT2 foot-and-mouth disease virus using chicken single-chain antibody fragments. Virus Res. (2012) 167:370–9. 10.1016/j.virusres.2012.05.026 22698877

[26] Maree FF Kasanga CJ Scott KA Opperman PA Chitray M Sangula AK. Challenges and prospects for the control of foot-and-mouth disease: an African perspective. Vet Med Res Rep. (2014) 5:119–38. 10.2147/VMRR.S62607 32670853 PMC7337166

[27] Davidson FL Crowther JR Nqindi J Knowles NJ Thevasagayam SJ Van Vuuren CJ. Antigenic analysis of SAT 2 serotype foot-and-mouth disease virus isolates from Zimbabwe using monoclonal antibodies. Epidemiol Infect. (1995) 115:193–205. 10.1017/S095026880005826X 7543860 PMC2271547

[28] Opperman P. Antigenic site determination on a SAT2 foot-and-mouth disease virus using a chicken antibody phage display library by P.A Opperman (thesis). University of Pretoria, Pretoria, South Africa (2013).

[29] Xie QC McCahon D Crowther JR Belsham GJ McCullough KC. Neutralization of foot-and-mouth disease virus can be mediated through any of at least three separate antigenic sites. J Gen Virol. (1987) (Pt 6):1637–47. 10.1099/0022-1317-68-6-1637 2438378

[30] Acharya R Fry E Stuart D Fox G Rowlands D Brown F. The three-dimensional structure of foot-and-mouth disease virus at 2.9 A resolution. Nature. (1989) 337:709–16. 10.1038/337709a0 2537470

[31] Dunn CS Samuel AR Pullen LA Anderson J. The biological relevance of virus neutralisation sites for virulence and vaccine protection in the guinea pig model of foot-and-mouth disease. Virology. (1998) 247:51–61. 10.1006/viro.1998.9175 9683571

[32] Köhler G Milstein C. Continuous cultures of fused cells secreting antibody of predefined specificity. Nature. (1975) 256:495–7. 10.1038/256495a0 1172191

[33] Willats WGT Phage display: practicalities and prospects. Plant Mol Biol. (2002) 50:837–54. 10.1023/A:1021215516430 12516857

[34] Pandey S Hybridoma technology for the production of monoclonal antibodies. Int J Pharmac Sci Rev Res. (2010) 1:88–94.

[35] Smith GP Petrenko VA. Phage display. Chem Rev. (1997) 97:391–410. 10.1021/cr960065d 11848876

[36] van Wyngaardt W Malatji T Mashau C Fehrsen J Jordaan F Miltiadou D. A large semi-synthetic single-chain Fv phage display library based on chicken immunoglobulin genes. BMC Biotechnol. (2004) 4:6. 10.1186/1472-6750-4-6 15059288 PMC406508

[37] Harmsen MM van Solt CB Fijten HPD van Keulen L Rosalia RA Weerdmeester K. Passive immunization of guinea pigs with llama single-domain antibody fragments against foot-and-mouth disease. Vet Microbiol. (2007) 120:193–206. 10.1016/j.vetmic.2006.10.029 17127019

[38] Yu Y Wang H Zhao L Zhang C Jiang Z Yu L. Fine mapping of a foot-and-mouth disease virus epitope recognized by serotype-independent monoclonal antibody 4B2. J Microbiol. (2011) 49:94–101. 10.1007/s12275-011-0134-1 21369985

[39] Fehrsen J van Wyngaardt W Mashau C Potgieter AC Chaudhary VK Gupta A. Serogroup-reactive and type-specific detection of bluetongue virus antibodies using chicken scFvs in inhibition ELISAs. J Virol Methods. (2005) 129:31–9. 10.1016/j.jviromet.2005.04.015 15946749

[40] Rakabe M Selection of chicken single-chain antibody fragments directed against recombinant VP7 of bluetongue virus (thesis). University of Pretoria, Pretoria, South Africa (2008). p. 1–111. 10.1080/09540105.2011.575122

[41] Sixholo J Van Wyngaardt W Mashau C Frischmuth J Du Plessis DH Fehrsen J. Improving the characteristics of a mycobacterial 16 kDa-specific chicken scFv. Biologicals. (2011) 39:110–6. 10.1016/j.biologicals.2011.01.007 21349739

[42] Nukarinen T Production and characterisation of scFv binders against selected Enteroviruses by T Nukarinen (thesis). University of Eastern Finland, Joensuu, Finland (2016).

[43] Knipe T Rieder E Baxt B Ward G Mason PW. Characterization of synthetic foot-and-mouth disease virus provirions separates acid-mediated disassembly from infectivity. J Virol. (1997) 71:2851–6. 10.1128/JVI.71.4.2851-2856.1997 9060641 PMC191410

[44] Doel TR Mowat GN. An international collaborative study on foot and mouth disease virus assay methods. 2. Quantification of 146S particles. J Biol Stand. (1985) 13:335–44. 10.1016/S0092-1157(85)80048-2 2997228

[45] Hall T BioEdit sequence alignment editor for windows 95/98/NT/XP/Vista/7. Nucleic Acids Sympos Ser. (1999) 41:95–8.

[46] Office International des Epizooties Principles and Methods of Validation of Diagnostic Assays for Infectious Diseases. Manual of Diagnostic Tests and Vaccines for Terrestrial Animals 2018 (2018).

[47] Lea S Abu-Ghazaleh R Blakemore W Curry S Fry E Jackson T. Structural comparison of two strains of foot-and-mouth disease virus subtype O1 and a laboratory antigenic variant, G67. Structure. (1995) 3:571–80. 10.1016/S0969-2126(01)00191-5 8590018

[48] Namatovu A Belsham GJ Ayebazibwe C Dhikusooka MT Wekesa SN Siegismund HR. Challenges for serology-based characterization of foot-and-mouth disease outbreaks in Endemic areas; identification of two separate lineages of serotype O FMDV in Uganda in 2011. Transbound Emer Dis. (2015) 62:522–34. 10.1111/tbed.12170 24118785

[49] van Rensburg HG Nel LH. Characterization of the structural-protein-coding region of SAT 2 type foot-and-mouth disease virus. Virus Genes. (1999) 19:229–33. 10.1023/A:1008140815045 10595414

[50] Ahmad ZA Yeap SK Ali AM Ho WY Alitheen NBM Hamid M. ScFv antibody: Principles and clinical application. Clin Develop Immunol. (2012) 2012:980250. 10.1155/2012/980250 22474489 PMC3312285

[51] Carmen S Jermutus L. Concepts in antibody phage display. Brief Funct Genomics Proteomics. (2002) 1:189–203. 10.1093/bfgp/1.2.189 15239904

[52] Hamers-Casterman C Atarhouch T Muyldermans S Robinson G Hamers C Songa EB. Naturally occurring antibodies devoid of light chains. Nature. (1993) 363:446–8. 10.1038/363446a0 8502296

[53] Williamson P Matthews R. Development of neutralising human recombinant antibodies to pertussis toxin. FEMS Immunol Med Microbiol. (1999) 23:313–9. 10.1016/S0928-8244(98)00151-5 10225291

[54] Pfaff E Thiel HJ Beck E Strohmaier K Schaller H. Analysis of neutralizing epitopes on foot-and-mouth disease virus. J Virol. (1988) 62:2033–40. 10.1128/JVI.62.6.2033-2040.1988 2835507 PMC253288

[55] Barnett PV Ouldridge EJ Rowlands DJ Brown F Parry NR. Neutralizing epitopes of type O foot-and-mouth disease virus. I. Identification and characterization of three functionally independent, conformational sites. J Gen Virol. (1989) 70 (Pt 6):1483–91. 10.1099/0022-1317-70-6-1483 2471811

[56] Parry NR Barnett PV Ouldridge EJ Rowlands DJ Brown F. Neutralizing epitopes of type O foot-and-mouth disease virus. II Mapping three conformational sites with synthetic peptide reagents. J Gen Virol. (1989) 70:1493–503. 10.1099/0022-1317-70-6-1493 2471812

[57] Mahapatra M Seki C Upadhyaya S Barnett PV La Torre J Paton DJ. Characterisation and epitope mapping of neutralising monoclonal antibodies to A24 Cruzeiro strain of FMDV. Vet Microbiol. (2011) 149:242–7. 10.1016/j.vetmic.2010.11.003 21144677

[58] Rieder E Baxt B Mason PW. Animal-derived antigenic variants of foot-and-mouth disease virus type A12 have low affinity for cells in culture. J Virol. (1994) 68:5296–9. 10.1128/JVI.68.8.5296-5299.1994 8035529 PMC236478

[59] Lawrence P Larocco M Baxt B Rieder E. Examination of soluble integrin resistant mutants of foot-and-mouth disease virus. Virol J. (2013) 10:2. 10.1186/1743-422X-10-2 23282061 PMC3547720

[60] Jackson T Sheppard D Denyer M Blakemore W King AMQ. The epithelial integrin αvβ6 is a receptor for foot-and-mouth disease virus. J Virol. (2000) 74:4949–56. 10.1128/JVI.74.11.4949-4956.2000 10799568 PMC110846

[61] Toth RL Harper K Mayo MA Torrance L. Fusion proteins of single-chain variable fragments derived from phage display libraries are effective reagents for routine diagnosis of potato leafroll virus infection in potato. Phytopathology. (1999) 89:1015–21. 10.1094/PHYTO.1999.89.11.1015 18944656

[62] Bittle JL Houghten RA Alexander H Shinnick TM Sutcliffe JG Lerner RA. Protection against foot-and-mouth disease by immunization with a chemically synthesized peptide predicted from the viral nucleotide sequence. Nature. (1982) 298:30–3. 10.1038/298030a0 7045684

[63] Pfaff E Mussgay M Böhm HO Schulz GE Schaller H. Antibodies against a preselected peptide recognize and neutralize foot and mouth disease virus. EMBO J. (1982) 1:869–74. 10.1002/j.1460-2075.1982.tb01262.x 6203738 PMC553124

[64] Jung Y Jeong JY Chung BH. Recent advances in immobilization methods of antibodies on solid supports. Analyst. (2008) 133:697–701. 10.1039/b800014j 18493668

[65] Qian W Yao D Yu F Xu B Zhou R Bao X. Immobilization of antibodies on ultraflat polystyrene surfaces. Clin Chem. (2000) 46:1456–63. 10.1093/clinchem/46.9.1456 10973890

[66] Kumada Y Ishikawa Y Fujiwara Y Takeda R Miyamoto R Niwa D. Efficient refolding and immobilization of PMMA-tag-fused single-chain Fv antibodies for sensitive immunological detection on a PMMA plate. J Immunol Methods. (2014) 411:1–10. 10.1016/j.jim.2014.05.015 24910412

[67] McCullough KC Crowther JR Butcher RN Alteration in antibody reactivity with foot-and-mouth disease virus (FMDV) 146S antigen before and after binding to a solid phase or complexing with specific antibody. J Immunol Methods. (1985) 82:91–100. 10.1016/0022-1759(85)90228-5 2993421

[68] Kumada Y Hamasaki K Shiritani Y Nakagawa A Kuroki D Ohse T. Direct immobilization of functional single-chain variable fragment antibodies (scFvs) onto a polystyrene plate by genetic fusion of a polystyrene-binding peptide (PS-tag). Analyt Bioanalyt Chem. (2009) 395:759–65. 10.1007/s00216-009-2999-y 19680637

[69] Kumada Y Hamasaki K Shiritani Y Ohse T Kishimoto M. Efficient immobilization of a ligand antibody with high antigen-binding activity by use of a polystyrene-binding peptide and an intelligent microtiter plate. J Biotechnol. (2009) 142:135–41. 10.1016/j.jbiotec.2009.03.011 19501265

[70] Torrance L Ziegler A Pittman H Paterson M Toth R Eggleston I. Oriented immobilisation of engineered single-chain antibodies to develop biosensors for virus detection. J Virol Methods. (2006) 134:164–70. 10.1016/j.jviromet.2005.12.012 16427706

[71] Wemmer S Mashau C Fehrsen J van Wyngaardt W du Plessis DH. Chicken scFvs and bivalent scFv-C(H) fusions directed against HSP65 of *Mycobacterium bovis*. Biologicals. (2010) 38, 407–414. 10.1016/j.biologicals.2010.02.002 20299243

[72] Hoogenboom HR Winter G. By-passing immunisation. Human antibodies from synthetic repertoires of germline VH gene segments rearranged *in vitro*. J Mol Biol. (1992) 227:381–8. 10.1016/0022-2836(92)90894-P 1404359

[73] Griffiths AD Malmqvist M Marks JD Bye JM Embleton MJ McCafferty J. Human anti-self antibodies with high specificity from phage display libraries. EMBO J. (1993) 12:725–34. 10.1002/j.1460-2075.1993.tb05706.x 7679990 PMC413258

[74] O'Connell D Becerril B Roy-Burman A Daws M Marks JD. Phage versus phagemid libraries for generation of human monoclonal antibodies. J Mol Biol. (2002) 321:49–56. 10.1016/S0022-2836(02)00561-2 12139932

[75] Mcgregor DP Molloy PE Cunningham C Harris WJ. Spontaneous assembly of bivalent single chain antibody fragments in *Escherichia coli*. Mol Immunol. (1994) 31:219–26. 10.1016/0161-5890(94)90002-7 8114767

[76] Griep RA Prins M van Twisk C Keller HJ Kerschbaumer RJ Kormelink R. Application of phage display in selecting *Tomato spotted wilt virus*-specific Single-Chain Antibodies (scFvs) for sensitive diagnosis in ELISA. Phytopathology. (2000) 90:183–90. 10.1094/PHYTO.2000.90.2.183 18944607

[77] Liu J Wei D Qian F Zhou Y Wang J Ma Y. pPIC9-Fc: a vector system for the production of single-chain Fv-Fc fusions in *Pichia pastoris* as detection reagents *in vitro*. J Biochem. (2003) 134:911–7. 10.1093/jb/mvg222 14769881

[78] Kerschbaumer RJ Hirschl S Kaufmann A Ibl M Koenig R Himmler G. Single-chain Fv fusion proteins suitable as coating and detecting reagents in a double antibody sandwich enzyme-linked immunosorbent assay. Analyt Biochem. (1997) 249:219–27. 10.1006/abio.1997.2171 9212874

[79] Chitray M Improvement of foot-and-mouth disease virus vaccines and diagnostics through structural design (thesis). University of Pretoria, Pretoria, South Africa (2018).

